# Integration of mark–recapture and acoustic detections for unbiased population estimation in animal communities

**DOI:** 10.1002/ecy.3769

**Published:** 2022-07-15

**Authors:** Crinan Jarrett, Daniel T. Haydon, Juan M. Morales, Diogo F. Ferreira, Francis Alemanji Forzi, Andreanna J. Welch, Luke L. Powell, Jason Matthiopoulos

**Affiliations:** ^1^ Institute of Biodiversity, Animal Health and Comparative Medicine, College of Medical Veterinary and Life Sciences University of Glasgow Glasgow UK; ^2^ Biodiversity Initiative Belmont Massachusetts USA; ^3^ Grupo de Ecología Cuantitativa, INIBIOMA‐CONICET Universidad Nacional del Comahue Bariloche Argentina; ^4^ CIBIO, Centro de Investigação em Biodiversidade e Recursos Genéticos, InBIO Laboratório Associado, Campus de Vairão Universidade do Porto Vairão Portugal; ^5^ BIOPOLIS Program in Genomics, Biodiversity and Land Planning CIBIO Vairão Portugal; ^6^ Partners for Sustainable Development Buea Cameroon; ^7^ Department of Biosciences Durham University Durham UK

**Keywords:** acoustic data, Afrotropics, Bayesian model, bird, data integration, hierarchical modeling, mark–recapture data, population size

## Abstract

Abundance estimation methods that combine several types of data are becoming increasingly common because they yield more accurate and precise parameter estimates and predictions than are possible from a single data source. These beneficial effects result from increasing sample size (through data pooling) and complementarity between different data types. Here, we test whether integrating mark–recapture data with passive acoustic detections into a joint likelihood improves estimates of population size in a multi‐guild community. We compared the integrated model to a mark–recapture‐only model using simulated data first and then using a data set of mist‐net captures and acoustic recordings from an Afrotropical agroforest bird community. The integrated model with simulated data improved accuracy and precision of estimated population size and detection parameters. When applied to field data, the integrated model was able to produce, for each bird guild, ecologically plausible estimates of population size and detection parameters, with more precision compared with the mark–recapture model. Overall, our results show that adding acoustic data to mark–recapture analyses improves estimates of population size. With the increasing availability of acoustic recording devices, this data collection technique could readily be added to routine field protocols, leading to a cost‐efficient improvement of traditional mark–recapture population estimation.

## INTRODUCTION

Evaluating trends in species abundance is central to questions of single species and whole ecosystem conservation. Hence, estimating the size of populations remains as critical as it is challenging. At the heart of the issue is that ecological sampling of organisms is rarely exhaustive, and therefore to estimate population size we need to also estimate probabilities of detection (Dorazio, 2014). Detectability may vary according to a wide range of variables, such as weather conditions or observer skill level. To help deal with imprecision and bias in data collection, there has been an upsurge in methods that combine different data sources to generate accurate and more precise estimates of species distribution and density (Fithian et al., 2015; Koshkina et al., 2017; Peel et al., 2019; Williams et al., 2017).

The concept of data integration (combining data types) relies heavily on complementarities between data sources. In general, different methods of data collection will suffer from different detection biases; for instance, it may be more likely that a species is detected by citizen science programs in areas with high human population density (Johnston et al., 2020), and it may be easier to detect animals on visual surveys in open compared to forested habitats (Rodrigues & Prado, 2018). Data integration helps deal with these biases, because shared biological parameters are estimated simultaneously from multiple data types that are not equally vulnerable to the same problems (Miller et al., 2019). Thus far, integrated models have largely focused on spatially explicit landscape‐level models of species distribution, often generated using large online data sets. Whether data integration methods will be effective in estimating abundance of species in smaller‐scale field‐generated data sets is less clear (Isaac et al., 2020).

Mark–recapture has been employed for population estimation across a broad range of taxa including birds, mammals and fish (Schwarz & Seber, 1999). While mark–recapture analyses can provide robust estimates of population size, they can also suffer from biases, for instance when environmental covariates affect both detectability and population size (Banks‐Leite et al., 2014; Oyster et al., 2018). Data integration methods can mitigate these problems and estimate the contributions of the different determinants of abundance and detection. In the case of mark–recapture, we could expect a benefit of combining these data with another type of data, ideally one collected using a method whose detectability is not influenced by the same variables.

Acoustic data could provide this opportunity. Acoustic recordings are usually collected using automated devices that are easy to operate, require low levels of effort (installation, collection, and storage), and can generate a wealth of data (Bradfer‐Lawrence et al., 2020). However, abundance estimation from acoustics alone, though feasible, can be complicated due to either double‐counting (overestimation) or saturation (underestimation) effects (Dawson & Efford, 2009; Doser et al., 2021). Additionally, to estimate abundance from acoustic data it is often necessary to know species' vocalization rates, which is typically not feasible. Combining mark–recapture with acoustics could result in improved estimates, partly because the factors affecting detectability of each method are different (Dawson & Efford, 2009). In a practical sense, simultaneously collecting both mark–recapture and acoustic data in the field is very achievable; mark–recapture protocols are already widespread and adding an automatic recording unit to these systems is logistically simple and inexpensive (Whytock & Christie, 2017).

A recent study by Doser et al. (2021) combined acoustic data with point counts to estimate the abundance of a bird species and found that the integrated model improved accuracy and precision of results. Here, we build on the ideas of Doser et al. (2021) and take the next natural steps, from point counts to mark–recapture data (as is common in, e.g., bird surveys) and from single species to whole communities. Our modeling framework integrates mark–recapture data and count data from acoustic recordings to estimate population size of a multi‐guild community. This method is widely applicable to any animal community for which mark–recapture and acoustic data are available. Our aim was to assess whether the addition of acoustic data to mark–recapture models improved accuracy and precision of population size estimates, and to illustrate the application of the method to a data set of bird communities in African agro‐ecosystems.

## METHODS

### Model overview and assumptions

Our model integrates mark–recapture and acoustic data into a joint likelihood, to estimate population size (N). Our focus was on exploring the trade‐offs and complementarities of the joint analysis of these two common data types, and we wished to isolate these issues from the wider problems of population change and emigration. Therefore, we assumed throughout that population size remained constant during each sampling period.

#### Determinants of population size

Throughout these analyses, we considered animal communities made up of several subgroups. We use the term “guilds” to describe taxonomic groups, which may be species but could also be functional groups, families, etc. We considered site as the discrete area covered by the sampling radius of our mist nets, and therefore the population size at site *j* is the number of animals whose home ranges overlap with this area. Importantly, we assumed that the detection radius of the acoustic recorder(s) was the same or smaller than the mist‐net sampling radius, so that the number of individuals detectable by acoustic recorders was proportional (but not necessarily equal) to the number of individuals detectable by mist nets. We assumed that the population size ($N_{\mathrm{ij}}$) of guild *i* at site *j* was Poisson distributed (but our method is easily adapted to other distributions) with expected number of animals per site $D_{\mathrm{ij}}$

(1) $$N_{\mathrm{ij}}∼\text{Poisson}\left(D_{\mathrm{ij}}\right)$$

And $D_{\mathrm{ij}}$ was modeled as a log‐linear function

(2) $$\log\left(D_{\mathrm{ij}}\right)=\sum_{q=0}^{Q_{f}}\nu_{\mathrm{iq}}X_{\mathrm{jq}}.$$

The linear predictor comprised $Q_{f}$ covariates, $X_{\mathrm{jq}}$, affecting population size, and their respective regression coefficients $\nu_{\mathrm{iq}}$, where *q* refers to the *q*th covariate (the intercept ($\nu_{i0})$ was included by setting $X_{j0}=1$).

#### Mark–recapture

Instead of dividing each survey event into arbitrary discrete time periods as is commonly done in mark–recapture studies (Schofield et al., 2018), we modeled the capture history of every animal as a homogeneous Poisson process (HPP) in continuous time. The HPP had a rate of $r_{\mathrm{ij}}$ captures per unit time (Xi et al., 2007), and the time period over which sampling occurred was $T_{j}$. The HPP process implies that the waiting time to first capture of an individual *k* of guild *I* at site *j* (*c*_*kij*_) follows an exponential distribution with mean $1/r_{\mathrm{ij}}$

(3) $$c_{\mathrm{kij}}∼\text{Exponential}\left(r_{\mathrm{ij}}\right).$$

By definition, if $c_{\mathrm{kij}}>T_{j}$, the animal was not detected. The probability of detection is then $P\left(c_{\mathrm{kij}}<T_{j}\right)=1-\exp\left(-r_{\mathrm{ij}}T_{j}\right)$. Therefore, the total number of first captures $n_{\mathrm{ij}}$ for the *i*th guild at the *j*th site was

(4) $$n_{\mathrm{ij}}∼\text{Binomial}\left(N_{\mathrm{ij}},1-\exp\left(-r_{\mathrm{ij}}T_{j}\right)\;\right).$$

After the animal was caught once, the total number of recaptures ($y_{\mathrm{kij}}$) in the remaining time (conditional on $c_{\mathrm{kij}}<T_{j}$) was a Poisson variate given by

(5) $$y_{\mathrm{kij}}∼\text{Poisson}\left(r_{\mathrm{ij}}\left(T_{j}-c_{\mathrm{kij}}\right)\right).$$

Capture rate, $r_{\mathrm{ij}}$, was modeled as a log‐linear function

(6) $$\log\left(r_{\mathrm{ij}}\right)=\sum_{q=0}^{Q_{h}}\rho_{\mathrm{iq}}W_{\mathrm{jq}}$$

comprising $Q_{h}$ covariates, $W_{\mathrm{jq}}$, and their respective regression coefficients $\rho_{\mathrm{iq}}$, with *q* referring to the *q*th covariate and where $\rho_{i0}$ is the intercept. This mark–recapture model assumes that capture rate did not vary between individuals of same guild, did not decline with consecutive captures, and marks were not lost. Additionally, we assumed instantaneous sampling (i.e., individuals were immediately available for sampling after capture).

#### Acoustics

We assumed that it was not possible to identify individuals from acoustic data (but see Dawson & Efford, 2009). Additionally, non‐automated counting of vocalizations over a whole community from acoustic recordings would require large amounts of processing time. Therefore, to simplify data extraction (Appendix S1), we considered a set of $L_{j}$ discrete listening periods each lasting *M* time units, during which guilds may be heard and thus recorded as present. We modeled vocalizations as a HPP in continuous time with rate $\lambda_{\mathrm{ij}}N_{\mathrm{ij}}$ per unit time, where $\lambda_{\mathrm{ij}}$ was a site/guild‐specific per‐capita vocalization detection rate. The probability that at least one vocalization was recorded in any given listening period was the probability that the time to the first vocalization was less than *M*. We modeled the total number of detections $a_{\mathrm{ij}}$ over $L_{j}$ listening periods as

(7) $$a_{\mathrm{ij}}∼\text{Binomial}\left(L_{j},1-\exp\left(\lambda_{\mathrm{ij}}N_{\mathrm{ij}}M\right)\;\right).$$

Vocalization rate, $\lambda_{\mathrm{ij}}$, was modeled as a log‐linear function

(8) $$\log\left(\lambda_{\mathrm{ij}}\right)=\sum_{q=0}^{Q_{r}}\psi_{\mathrm{iq}}G_{\mathrm{jq}}$$

comprising $Q_{r}$ covariates, $G_{\mathrm{jq}}$, and their respective regression coefficients $\psi_{\mathrm{iq}}$, with *q* referring to the *q*th covariate, and $\psi_{i0}$ the intercept.

We assume that the probability of capturing an individual bird is independent of the probability of it being detected by the acoustic recorder, and consequently the mark–recapture and acoustic models are independent, both conditional on the true latent population size $N_{\mathrm{ij}}$ (Miller et al., 2019). We fit our models using Bayesian inference with the JAGS 4.3.0 software (Plummer, 2017) executed using the runjags package (Denwood, 2016) in the R statistical computing environment (R Core Team, 2020). For each model, we ran three chains of 100,000 iterations with a burn‐in period of 5000 iterations. Model convergence was assessed by visually inspecting chains and with the Gelman‐Rubin R‐hat diagnostic, with convergence presumed when *R* < 1.1.

### Simulation study

To assess whether the integrated model produced more accurate and precise estimates compared with single‐data‐set models, we compared it with a model that used just mark–recapture data (Equations (1), (2), (3), (4), (5), (6)). We applied each model to simulated data from 20 sites each assumed to contain three guilds (labeled *A*, *B*, and *C*), and to have been visited twice. This represents a minimally realistic design for a mark–recapture study, given the number of guilds and parameters involved. We included several environmental covariates: the first was a site‐specific covariate that affected both population size and capture rate (Equations 2 and 6). We added this covariate because previous models have encountered identifiability issues when retrieving covariates that affect both population size and detectability (Fithian et al., 2015; Simmonds et al., 2020). The second covariate affecting population size was shared across guilds and sites but varied with visit (Equation 2). We generated data using the model statements above and used the same priors for both models (see Table 1 for parameter values and priors). At each site, capture period $T_{j}$ was set to 6 h, and we assumed 20 listening periods ($L_{j}$) each lasting 0.03 hours (2 min; *M*). For both models we examined accuracy and precision by recording the mean and 95% Bayesian credible intervals (BCIs; calculated for the highest posterior density intervals) from each posterior. Additionally, we performed model validation to assess the effects of a range of data‐generating parameters on model performance, and an exploration of trade‐offs between acoustic and mark–recapture effort (Appendix S1). With this exploration we aimed to help users optimize field survey effort allocation to maximize accuracy and precision of population size estimates.

**TABLE 1:** For each parameter in the model, true value used to simulate data, prior (standard deviation [SD]), and mean (95% Bayesian credible intervals) of parameter estimates from model with only capture–recapture data and integrated model.

Parameter	Description	Simulation value	Prior (SD)	Capture–recapture	Integrated
ν_*A*0_	Intercept of linear predictor of population size for species *A*	5.00	6.00 (0.82)	4.85 (4.26, 5.52)	4.88 (4.55, 5.25)
ν_*B*0_	Intercept of linear predictor of population size for species *B*	3.00	6.00 (0.82)	3.36 (2.61, 4.21)	3.34 (2.72, 3.94)
ν_*C*0_	Intercept of linear predictor of population size for species *C*	4.50	6.00 (0.82)	4.77 (3.94, 5.65)	4.86 (4.04, 5.70)
ν_1_	Effect of site‐level covariate *X*_*j*1_ on population size	0.01	0.00 (1.00)	0.01 (0, 0.02)	0.01 (0.01, 0.01)
ν_2_	Effects of visit‐level covariate *X*_2_ on population size	0.15	0.00 (0.20)	0.11 (−0.01, 0.23)	0.11 (0.01, 0.21)
ρ_*A*0_	Intercept of linear predictor of capture rate for species *A*	−4.00	0.00 (3.16)	−3.82 (−4.49, −3.22)	−3.86 (−4.23, −3.5)
ρ_*B*0_	Intercept of linear predictor of capture rate for species *B*	−3.50	0.00 (3.16)	−3.75 (−4.64, −2.98)	−3.75 (−4.43, −3.10)
ρ_*C*0_	Intercept of linear predictor of capture rate for species *C*	−4.80	0.00 (3.16)	−5.04 (−5.95, −4.19)	−5.13 (−5.99, −4.28)
ρ_1_	Effect of site‐level covariate *W*_*j*1_ on capture rate	−0.01	0.00 (1.00)	−0.01 (−0.02, 0)	−0.01 (−0.02, −0.01)
Ψ_*A*0_	Intercept of linear predictor of vocalization rate for species *A*	−7.00	−5.00 (2.00)	…	−6.9 (−7.26, −6.56)
Ψ_*B*0_	Intercept of linear predictor of vocalization rate for species *B*	−5.00	−5.00 (2.00)	…	−5.35 (−5.98, −4.74)
Ψ_*C*0_	Intercept of linear predictor of vocalization rate for species *C*	−6.00	−5.00 (2.00)	…	−6.38 (−7.24, −5.56)

*Note*: The distribution of the priors was normal, in every case. See Equations ((1), (2), (3), (4), (5), (6), (7), (8)) for further details.

### Case study

We used mist netting and acoustic data from birds in 32 sites in Cameroon, collected over 4 years (2017–2020; for details see Appendix S1 and Jarrett et al., 2021). Sampling events occurred during either the dry season or the wet season.

#### Model structure

Population size of each of the six bird groups at each sampling event *z* (Appendix S1) was modeled with a guild‐specific intercept ($\nu_{i0}$) and two covariates ($Q_{f}=2)$: a guild‐specific effect of canopy cover (continuous variable, centered and standardized) on population size ($\nu_{i1}X_{j1}$) and a seasonal categorical covariate shared between guilds ($\nu_{2}X_{z2}$, where $X_{z2}=1$ if Dry and 0 otherwise; Equation 9)

$$N_{\mathrm{ijz}}∼\text{Poisson}\left(D_{\mathrm{ijz}}\right)$$

(9) $$\log\left(D_{\mathrm{ijz}}\right)=\nu_{i0}+\nu_{i1}X_{j1}+\nu_{2}X_{z2}.$$

We modeled total captures for each sampling unit as in Equation (4) and individual bird capture histories with Equation (5). We considered capture rate $r_{\mathrm{ij}}$ to be guild specific and to vary between sites according to canopy cover ($Q_{h}=1;$ Equation 10), and we considered vocalization detection rate to be guild specific but not influenced by detection covariates ($Q_{r}=0;$ Equation 11; Appendix S1)

(10) $$\log\left(r_{\mathrm{ij}}\right)=\rho_{i0}+\rho_{1}W_{j1}$$

(11) $$\mathrm{Log}\left(\lambda_{i}\right)=\psi_{i0}.$$

We modeled number of vocalizations with Equation (7).

Priors used for model fitting were normally distributed (Jarrett, 2022). To compare the performance of integrated versus single‐data‐set models, we fit a model that used only mark–recapture data. For both models we recorded parameter estimates and BCIs to assess posterior precision of one method relative to another.

## RESULTS

### Simulation study

**FIGURE 1:**
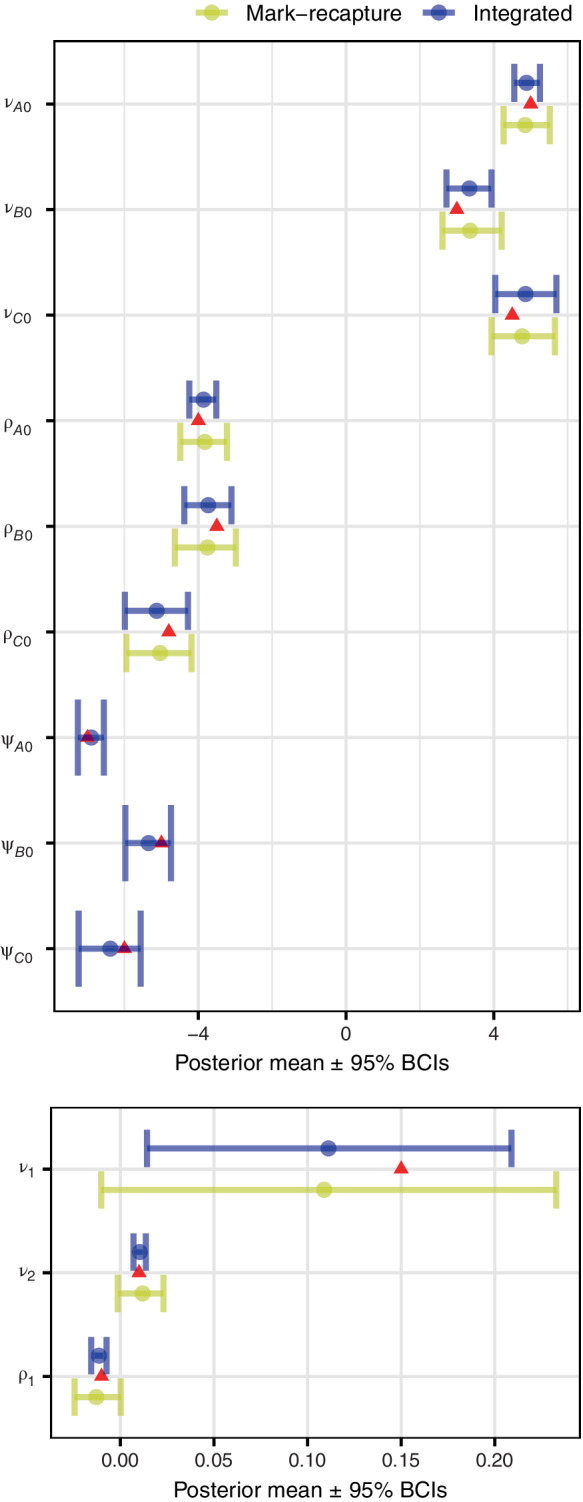
Mean of posterior distribution (and 95% Bayesian credible intervals [BCIs]) for population size and detection parameters from mark–recapture only model and integrated model under simulation. Only the integrated model (with acoustic data) calculated vocalization rates. The triangles represent the simulation values. For parameter definitions see Table 1.

The results from testing our model on simulated mark–recapture and acoustic data demonstrated that overall, the integrated model more accurately estimated all relevant parameters: capture rates, vocalization rates, detection covariate and covariates affecting population size (Figure 1 and Table 1). Compared with the mark–recapture model, the estimates of population size from the integrated model were more accurate and precise (Jarrett, 2022). The simulation also confirmed that the parameter estimates from the integrated model were more accurate and more precise (Figure 1; Table 1). The single‐data‐set mark–recapture model estimated with low precision the coefficients corresponding to the covariate that affected both population size and capture rate ($\nu_{1}$ and $\rho_{1}$). For these two coefficients, the integrated model was more than two times more precise than the mark–recapture model (Figure 1).

### Case study

**FIGURE 2:**
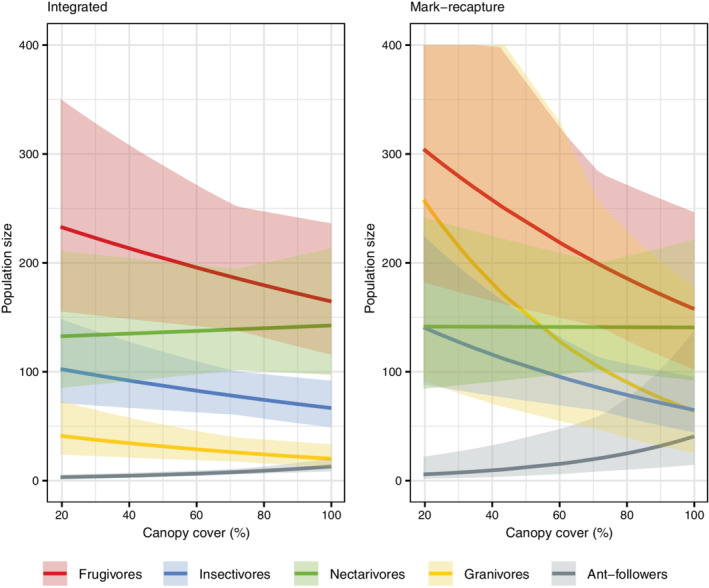
Population size (mean and 95% Bayesian credible intervals [BCIs]) of bird guilds with canopy cover in Cameroonian cocoa farms and forest in the dry season, estimated from both mark–recapture model and integrated model. The guild “other” was excluded from plots for the purpose of visual clarity.

We fit our integrated and single‐data‐set models to mist‐netting and acoustic data from Cameroonian cocoa farms and forest sites. Compared with the mark–recapture only model, the integrated model produced more precise estimates for model parameters and population size (Figure 2; Jarrett, 2022). The integrated model estimated with ∼1.5 times more precision parameters $\nu_{i1}$, which quantify the effect of canopy on population size of each guild. Estimated population sizes of bird guilds were 165–233 for frugivores, 67–102 for insectivores, 133–143 for nectarivores, 3–13 for ant‐followers, 20–41 for granivores, and 67–83 for other. The effect of canopy cover on abundance was different between groups (Figure 2); frugivores, insectivores, and granivores decreased with increasing canopy cover, while ant‐followers showed the opposite trend. Nectarivores and other birds were largely unaffected by canopy cover (Figure 2).

## DISCUSSION

The improved accuracy and precision of parameter estimates resulting from our integrated model compared with the simpler model match previous findings using different types of data (Doser et al., 2021; Fithian et al., 2015; Koshkina et al., 2017; Pacifici et al., 2017; Peel et al., 2019). However, the superior performance of integrated models should not be taken for granted, especially in scenarios like our own, where sample sizes are relatively small and there is overlap between detection and environmental covariates (Simmonds et al., 2020). Simmonds et al. (2020) found that, when combining simulated presence‐only (PO) and presence–absence (PA) data, an integrated model only outperformed a simple model (just PA) beyond a certain sample size threshold. Additionally, they found that the PA‐only model was more accurate in predicting an environmental covariate if that covariate also influenced detection probability (Simmonds et al., 2020). In contrast, also using simulated PA and PO data, Peel et al. (2019) found little influence of sample size on accuracy or precision of the estimates from an integrated model. Overall, it appears that the effectiveness of integrated models at estimating parameters is variable and dependent on specific characteristics of the data used.

Doser et al.'s (2021) recent study deftly integrated point counts and acoustic surveys to estimate the abundance of a single bird species. They found that the integrated model produced more accurate and precise results compared with single‐data‐set models. Aside from the key difference in data types used (count versus mark–recapture) and the single‐ versus multi‐guild element, there were several additional differences between our approach and that of Doser et al. (2021). First, our model allows for covariates that are specific to each sampling instance, and therefore does not assume that populations stay constant between visits. Second, our formulation in continuous time allows for more flexibility in sampling intervals and covariates. Third, the processing of acoustic data was undertaken differently (semi‐automatic clustering algorithm versus manual identification), resulting in potential false positives in Doser et al.'s (2021) study but not in ours. Finally, Doser et al. (2021) did not include covariates that simultaneously affected detectability and population size. We conjecture that mark–recapture data may provide a significant advantage when it comes to estimating population size given these confounding covariates.

When applied to a real‐life scenario of bird populations, our integrated model produced relatively precise and ecologically plausible estimates. The estimated population size for the different feeding guilds were consistent with other studies from the tropics (Newmark, 2006), and the relative abundance of each guild matched previous knowledge from the system (Jarrett et al., 2021). The largest difference between the integrated and mark–recapture models was in the population size estimate for granivores, likely because granivores were not commonly caught in mist‐nets, yet had high vocalization rates, and therefore acoustic data for this species were rich.

The integrated model benefitted from borrowed strength between species, both in the observation and process components. In our observation model, species shared the effect of canopy cover on capture rates and, in the process model, species shared a seasonal trend. This is a small example of strength borrowing in a multi‐species framework, an approach carried out at much greater depth by Ovaskainen and Abrego (2020), among others. An additional benefit of our integrated model is that it provides potentially valuable information on species' vocalization rates.

To test the effectiveness of integrating data, we wished to remove the additional confounding factors of demographic processes. We therefore assumed that the population remained closed within each sampling period. Given the small sampling intervals used in the survey (6 h), this assumption is reasonable, but a natural extension of our model would be to consider longer sampling periods over which demographic processes occur. Additional extensions to this model could consider longitudinal effects such as capture shyness, which can be a common phenomenon in active trapping methods such as mist‐netting (Marques et al., 2013), and daily fluctuations in bird activity (especially vocalization). Despite these simplifying assumptions, our model provides a practical and expandable way to improve estimates of population size for small‐scale field studies. In general, adding an acoustic recording protocol to field surveys requires low effort and is relatively inexpensive (Bradfer‐Lawrence et al., 2020; Whytock & Christie, 2017). The addition of acoustic data may be especially beneficial when mark–recapture effort is limited (e.g., small number of visits) or when capture rates are low (Appendix S1). While identification of species from acoustic recordings can be a time‐consuming bottleneck, the increase in popularity of acoustic methods is resulting in more and more tools that help with this process (Darras et al., 2020).

In conclusion, the combination of acoustic and mark–recapture data offers an opportunity for more accurate and precise estimates of population size. This method can be applied to any taxa for which these data types are available, including birds, bats, cetaceans, and amphibians. To achieve accurate estimates of population size, we should move towards a modeling approach that accounts for possible biases and makes the most of available data.

## CONFLICT OF INTEREST

The authors declare no conflict of interest.

## Supporting information


Appendix S1

## Data Availability

Data and code (Jarrett, 2022) are provided via the following link: https://doi.org/10.6084/m9.figshare.15825111.v1.
