# Supplementary material for: Integration of mark–recapture and acoustic detections for unbiased population estimation in animal communities
Source: Ecology. 2022 Jul 15;103(10):e3769. doi: 10.1002/ecy.3769 (PMC9787363; doi:10.1002/ecy.3769)
Supplement: Supplementary file 1 — Appendix S1 [file ECY-103-e3769-s001.pdf]

Ecology

Integration of mark–recapture and acoustic detections for unbiased population estimation in animal communities

Crinan Jarrett, Daniel T. Haydon, Juan M. Morales, Diogo F. Ferreira, Francis Alemanji Forzi, Andreanna J. Welch, Luke L. Powell, and Jason Matthiopoulos

SUPPLEMENTARY MATERIALS: APPENDIX S1

METHODS

General survey design

We assumed a survey design consisting in any number of survey events at each site j. The population size N_{ij} was not assumed to stay constant between events. The number of times each individual animal was captured within a survey event constituted its capture history.

Effort analysis

We investigated the effect of sampling effort (both in mist-netting and acoustic recording) on model performance. Using the same population size and detection parameters as in the simulation study, we generated data corresponding to a range of 1–30 sampling hours (at intervals of 5 hrs) both for mist-netting and acoustic recordings. For mist-netting, the effort hours corresponded to the actual hours of mist-netting. For acoustic recording, as processing the recordings can be time-consuming, we assumed that for every 1 hr of recording we would need 2 hrs of processing (this is a conservative estimate, our experience is more in the range of 1.4 hrs of processing per recording hour). In other words, the actual field sampling hours used in the effort analysis were 1–30 for mist-netting and 1–10 for acoustic recording. We generated data for 36 combinations of these values and fit the integrated model to the resulting datasets. For each combination of values, we ran the model with three chains of 5,000 iterations with a burn-in period of 5,000 iterations and a thinning rate of 10.

We evaluated the results in terms of accuracy and precision: we examined accuracy by calculating the % bias of the posterior mean (i.e. $100 \times (Estimated\ mean - True\ mean)/True\ mean$) and we considered precision as the coefficient of variation (i.e. SD/Mean) of the posterior distribution. We used the coefficient of variation (CV) to measure precision (rather than BCIs as above) to make the measures comparable across different scenarios.

Model verification

To understand the sensitivity of the integrated model to variation in the main data generating parameters (linear predictors for capture and vocalization rate ρ_{i0} and ψ_{i0}), we simulated 121 combinations of parameters ρ_{i0} and ψ_{i0} ranging from –8 to –2 and –3.9 to 2 respectively, corresponding to rates in the range ~0 and 0.14 per capita captures per hour, and 0.02 and 8 detected vocalizations per hour. From each parameter combination we simulated 100 datasets, resulting in a total 12100 datasets. For this analysis, we used a simplified version of our simulation, looking at one site containing one guild, removing the covariates affecting N, ρ_{10} and ψ_{10}. We set the underlying population size *N* to be 50 in every case. For model fitting we set normally distributed priors N(0, 4.5) and N(–5, 4.5) for ρ_{10} and ψ_{10} respectively (mean and SD), and N(4.4, 4.5) for ν_{11}. For each combination of parameters, we ran the model with three chains of 5,000 iterations with a burn-in period of 5,000 iterations and a thinning rate of 10.

We evaluated the results in terms of accuracy and precision: we calculated accuracy as a percentage (as above) and we considered precision as the CV.

Case study

Field methods

Mist-netting and acoustic data were collected from birds in 28 cocoa farms and 4 primary forest sites in Cameroon over 4 years (2017–2020). Each of the 32 sites was visited between

2 and 6 times, in two different seasons. Sites were separated by at least 500 m and farms were at least 1.5 ha. Mature forest plots were in the Dja Faunal Reserve (3.19 N 12.81 E) and were at least 1 km from forest edge, had a closed canopy and no logging activity. Sites had varying canopy cover, which we predicted could influence both capture rate and population size. In cocoa farms, canopy cover is an indication of farm management; more intensively managed farms tend to have open canopies, whilst traditional agroforest farms have closed canopies.

At each site 20 12 x 3 m mist-nets (30 mm mesh) were set up, placed in a "T", "L" or "+" layout to fit the site boundaries (for farms). They were opened for 6 hrs (~6.20 am to 12.20 pm; $T_j = 6$), during which captured individuals were identified, ringed and then released. The Handbook of the Birds of the World (del Hoyo et al., 2019) was used to classify each species according to its primary food type, resulting in 6 mutually exclusive categories: insectivores, frugivores, nectarivores, ant-followers, granivores and other (including carnivores and piscivores; Jarrett et al., 2021). Though the categorization of species into guilds may mask some variability between species, it also makes parameter estimation a lot more feasible given the low number of captures for some species. We excluded any birds caught outside the 6hr sampling period. We considered recaptures as birds caught more than once during the same visit, but we excluded recaptures caught within 20 mins of release as this could indicate birds that flew straight back into nets due to disorientation or stress.

On the same day as the mist-netting, one automatic recording unit (ARU; Song Meter SM4, Wildlife Acoustics) was set up approximately at the center of mist-net transect, and programmed to record 6:30–6:40 AM and 7:30–7:40 AM. Due to the short time window over which acoustic data were extracted, we did not expect vocalization rate to vary temporally (as would be commonly expected if recordings covered e.g., whole day). The 20 min period was then divided into 1 min intervals ($L_j = 20$, $M = 0.03$; Equation 8), during which each species was recorded either as present (when a call was heard) or absent. The acoustic data

processing was done manually by one listener. The use of intervals was to facilitate data extraction from continuous recordings. We clumped species into the same 6 guilds applied to mist-net captures, and we excluded from the dataset any species that had never been caught in mist-nets (e.g., canopy species).

To measure canopy cover, photographs were taken at 10 locations in each site (corresponding to the center of every second mist-net), spaced out by 24 m and at minimum 50 m from farm edge. Photographs were taken using a camera with a fish-eye lens on an extendable pole (5 m) in order to extend above the cocoa trees growing in the understory. Using the software ImageJ (Schneider et al., 2012), we converted the photographs to black and white, and then calculated the percentage of black (vegetation) in each photograph. The shade cover value used was a mean of the 10 pictures. Sites were on a gradient of canopy cover, with values ranging from 19.6% to 100%.

RESULTS

Effort analysis

Accuracy was highest at >20 mist-netting hrs, with little effect of listening effort. However, at <10 mist-netting hours, increasing listening effort improved accuracy (Fig. S1). A similar trend was true for precision (Fig. S1). Models with <5 mist-netting hours consistently failed to converge, and models with <10 mist-netting hours converged only when there were more than 5 listening hours (Fig. S1).

Model verification

Accuracy in the mean of the posterior distributions for N, ρ_0 and ψ_0 was affected by the parameter values given to ρ_0 and ψ_0 in the simulation. The mean of the posterior distributions deviated from the given value by a maximum of –47.3%, 24.9%, and 25.3% for N, ρ_{10} and ψ_{10} respectively (Fig. S2a-c). Accuracy in the mean of the posterior distribution for N increased with increasing values of ρ_{10} and ψ_{10}, more steeply with the former. N was

consistently underestimated, likely due to the priors for the detection rate parameters; these were centered around 0, and an overestimation of ρ_{10} and ψ_{10} would result in an underestimation of population size. The ability of the model to retrieve parameter ρ_{10} remained relatively similar across the range of parameter values, whilst the accuracy in the estimation of ψ_{10} decreased at high values of ψ_{10}. Both parameters were consistently overestimated when at low values, likely due to the influence of their priors (centered at 0).

In terms of precision, the CV of the posterior distributions of *N*, ρ_{10} and ψ_{10} had maximum values of 0.68, 0.37 and 0.49, respectively (Fig. S2d-f). Precision in the estimate for *N* was lowest when both ρ_{10} and ψ_{10} were small, and a similar pattern was true for the estimation of ρ_{10}. For ψ_{10}, precision was consistently high until approximately –3.5, after which it decreased. All parameter combinations resulted in model convergence.

Case study

Abundance was ~1.3 times higher in the dry season compared with the wet season. Capture rate decreased with increasing canopy cover and vocalization detection rate ranged from 0.003 in nectarivores to 0.02 in granivores.

Figure S1. Accuracy (a) and precision (b) of posterior distributions for bird population size (N) across different effort scenarios, and model convergence across those same scenarios. We calculated accuracy (labelled 'diff.') of the posterior mean as $100 \times (Mean - Truth)/Truth$), and we considered precision as the coefficient of variation (CV) of the posterior distribution. We considered that a model achieved convergence if the Gelman-Rubin R-hat diagnostic for all model parameters was <1.1.

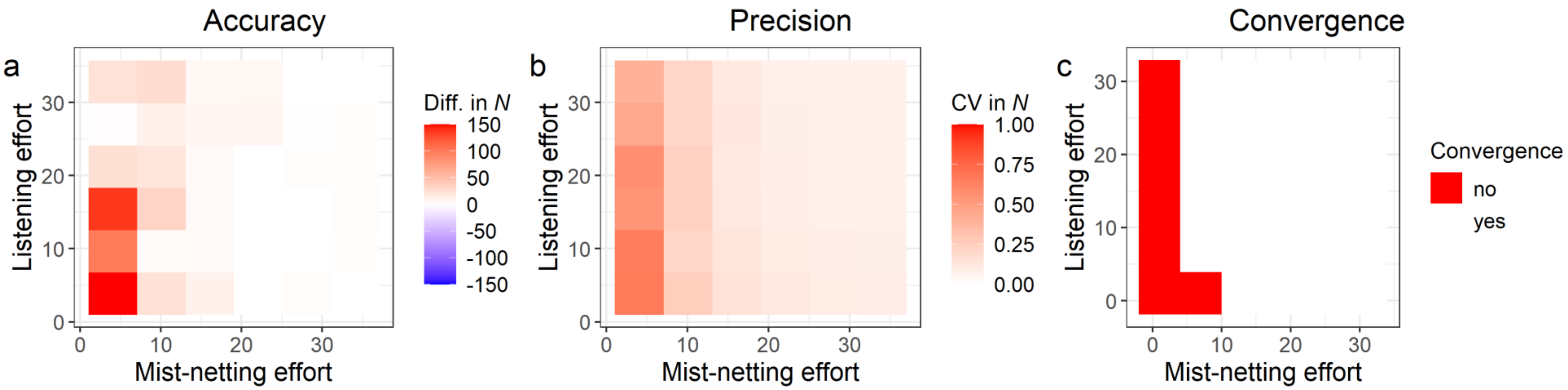

Figure S2. Accuracy (a – c) and precision (d – f) of posterior distributions for bird population size (N), capture rate (ρ_{10}) and vocalisation detection rate (ψ_{10}) from 1150 simulations generated using varying values for ρ_{10} and ψ_{10}, with N = 50, and replicated 10 times. We calculated accuracy (labelled 'diff.') of the posterior mean as $100 \times (Mean - Truth)/Truth$), and we considered precision as the coefficient of variation (CV) of the posterior distribution.

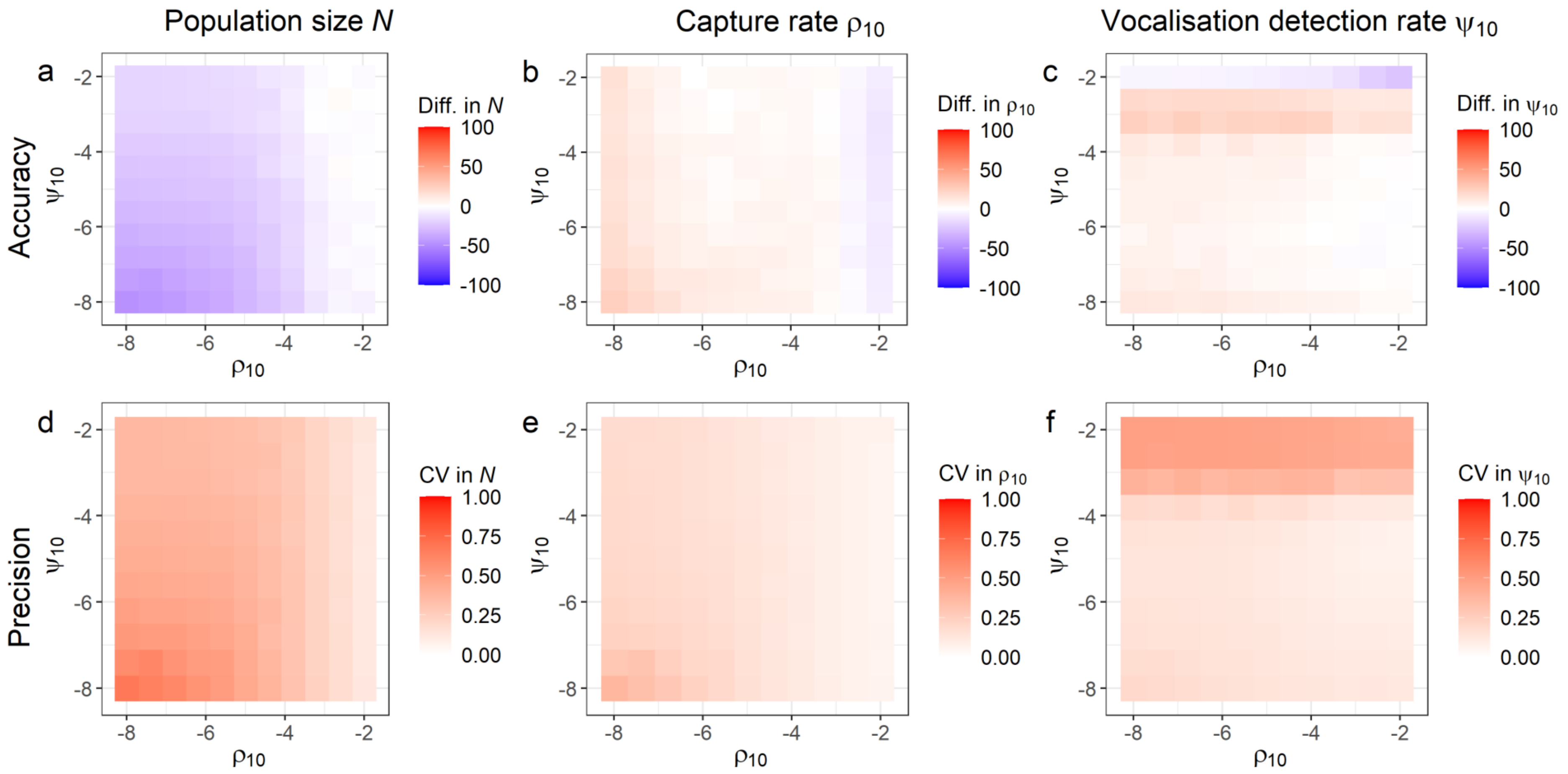

REFERENCES

del Hoyo, J., Elliott, A., Sargatal, J., Christie, D. A., & Kirwan, G. (2019). *Handbook of the Birds of the World Alive*. Lynx Edicions.

Jarrett, C., Smith, T. B., Claire, T. T. R., Ferreira, D. F., Tchoumbou, M., Elikwo, M. N. F., Wolfe, J., Brzeski, K., … Powell, L. L. (2021). Bird communities in African cocoa agroforestry are diverse but lack specialised insectivores. *Journal of Applied Ecology*, *58*(6), 1237–1247. https://doi.org/10.1111/1365-2664.13864

Schneider, C. A., Rasband, W. S., & Eliceiri, K. W. (2012). NIH Image to ImageJ: 25 years of image analysis. *Nature Methods*, *9*(7), 671–675.
